# Modelling the effects of standard prognostic factors in node-positive breast cancer

**DOI:** 10.1038/sj.bjc.6690279

**Published:** 1999-04

**Authors:** W Sauerbrei, P Royston, H Bojar, C Schmoor, M Schumacher, the German Breast Cancer Study Group (GBSG)

**Affiliations:** Institute of Medical Biometry and Medical Informatics, University of Freiburg, Stefan-Meier-Strasse 26, D-79104, Germany; Department of Medical Statistics and Evaluation, Imperial College School of Medicine, Hammersmith Hospital, Ducane Road, London, W12 0NN, UK; Department of Chemical Oncology, University of Düsseldorf, Germany

**Keywords:** breast cancer, prognostic factors, regression modelling, variable transformation, fractional polynomials

## Abstract

Prognostic models that predict the clinical course of a breast cancer patient are important in oncology. We propose an approach to constructing such models based on fractional polynomials in which useful transformations of the continuous factors are determined. The idea may be applied with all types of regression model, including Cox regression, the method of choice for survival-time data. We analyse a prospective study of node-positive breast cancer. Seven standard prognostic factors – age, menopausal status, tumour size, tumour grade, number of positive lymph nodes, progesterone and oestrogen receptor concentrations – were investigated in 686 patients, of whom 299 had an event for recurrence-free survival and 171 died. We determine a final model with transformations of prognostic factors and compare it with the more traditional approaches using categorized variables or assuming a straight line relationship. We conclude that analysis using fractional polynomials can extract important prognostic information which the traditional approaches may miss. © 1999 Cancer Research Campaign


*British Journal of Cancer* (1999) **79**(11/12), 1752–1760
© 1999 Cancer Research Campaign
Article no. bjoc.1998.0279

### Modelling the effects of standard prognostic factors in node-positive breast cancer

**W Sauerbrei[1], P Royston[2], H Bojar[3], C Schmoor[1], M Schumacher[1] and the German Breast Cancer Study Group (GBSG)**

[1]Institute of Medical Biometry and Medical Informatics, University of Freiburg, Stefan-Meier-Strasse 26, D-79104, Germany; [2]Department of Medical Statistics and Evaluation, Imperial College School of Medicine, Hammersmith Hospital, Ducane Road, London W12 0NN, UK; [3]Department of Chemical Oncology, University of Düsseldorf, Germany

**Summary** Prognostic models that predict the clinical course of a breast cancer patient are important in oncology. We propose an approach to constructing such models based on fractional polynomials in which useful transformations of the continuous factors are determined. The idea may be applied with all types of regression model, including Cox regression, the method of choice for survival-time data. We analyse a prospective study of node-positive breast cancer. Seven standard prognostic factors – age, menopausal status, tumour size, tumour grade, number of positive lymph nodes, progesterone and oestrogen receptor concentrations – were investigated in 686 patients, of whom 299 had an event for recurrence-free survival and 171 died. We determine a final model with transformations of prognostic factors and compare it with the more traditional approaches using categorized variables or assuming a straight line relationship. We conclude that analysis using fractional polynomials can extract important prognostic information which the traditional approaches may miss.

**Keywords:** breast cancer; prognostic factors; regression modelling; variable transformation; fractional polynomials

Prognostic models that predict the clinical course of a breast cancer patient and provide a rationale for her treatment are of central importance in oncology. Despite many projects and hundreds of papers in the last 2 decades only the nodal status is equivocally considered by all study groups as a strong factor in patients without metastases ($M_0$). Even the prognostic value of ‘standard factors’ such as tumour size, tumour grade, histologic type, oestrogen (ER) and progesterone receptor (PR) status, menopausal status and age is still controversial. During the last few years many new factors (clinicopathological, biological, molecular) have been investigated resulting in more than 100 proposed factors, most of them controversial. In addition to laboratory and clinically related problems in obtaining objective and reproducible measurements of a new factor, heterogeneity in patient populations and treatment or limitations of follow-up, a substantial part of this controversy concerns statistical aspects such as inadequate sample sizes, inadequate use of statistical methods and difficulties in comparing multivariable models with different factors or different categorizations of the factors (Simon and Altman, 1994).

In cancer clinical trials, where survival or recurrence-free survival (RFS) time is often the primary outcome variable, the statistical analysis is usually performed with the Cox proportional hazards model (Cox, 1972) where the effects of several prognostic factors on the risk of death are modelled simultaneously. The question arises as to how the factors should be included in the model, i.e. which form of relationship is to be assumed between a factor and the outcome variable. For factors like menopausal status (pre, peri, post) or tumour grade (I, II, III) with only two or a few levels, no major modelling difficulties arise. Binary variables can be used to indicate the membership of the patient to the corresponding level and the relative risks between the levels of the factor may be estimated in standard fashion.

Problems arise for continuous prognostic factors with many different values, e.g. age in years or tumour size in mm. Usually, such factors are included in the statistical model in their original form. This procedure implicitly assumes that the effect on the risk of death is log-linear, i.e. that the log relative risk increases or decreases linearly as the value of a factor increases. Obviously, this assumption may be wrong for some continuous factors, and mis-specification of the functional form may lead to wrong conclusions.

One common strategy to circumvent the assumption of a linear effect is to convert the continuous predictor into categorical factors by grouping patients into two or more groups. This enables researchers to avoid strong assumptions about the relation between the factor and risk, but at the expense of throwing away information. The information loss is greatest with only two groups, but the approach is widely used, e.g. for factors such as oestrogen and progesterone receptor (negative vs positive) or S phase fraction (low vs high). For S phase fraction, for instance, many different cutpoints are used; Altman et al (1994) listed 19 found in the literature and several methods for defining the cutpoint. It is often unclear which cutpoints to use. Sometimes patients are divided into two groups at the median value, but there is no a priori reason to assume that half of the patients are at higher risk (Simon and Altman, 1994). Often the cutpoint is used that gives the best separation of the patients into groups with different prognosis by repeating the analysis with all possible cutpoints. However the *P*-value of the test using the ‘best’ cutpoint is invalid without an

*Received 12 January 1998*
*Revised 11 August 1998*
*Accepted 20 August 1998*

*Correspondence to:* W Sauerbrei appropriate adjustment for the other tests, and the estimated effect of the factor is biased (Altman et al, 1994).

Tumour size is often analysed using three categories (≤ 20 mm, 21–50 mm, > 50 mm). For the most important factor, the number of involved lymph nodes, the categories 0, 1–3, 4–9 and ten or more are used. The effect of age is investigated in many different ways: sometimes just as a binary variable comparing young vs old, where many different cutpoints are in use, or with three categories or using 5- or 10-year intervals. Based on these categorized data, estimates of survival rates are compared in a univariate way, or multivariable models are developed using binary variables to represent the different categories. The serious problems with univariate analyses of prognostic factors are well known. The multivariable approach may lead to simple and interpretable models, but the loss of power because of categorization (Palta and Amini, 1985; Lagakos, 1988; Schmoor and Schumacher, 1997) may result in the analyst failing to notice an important factor. For a continuous variable the functional relationship with the risk of death is represented as a step-function, which can give only a rough idea about the true relationship; it is unlikely that the risk suddenly changes as one crosses a cutpoint.

In this paper we use a different approach for the analysis of the effect of continuous factors. It is based on fractional polynomials (FP) and was recently proposed to investigate the functional form of the effect of continuous factors in a systematic way (Royston and Altman, 1994; Sauerbrei and Royston, 1998). The new procedure is a flexible tool making use of the full information available in the data. It does not assume a linear relationship. Instead, for each factor the functional effect on the risk of death is assessed from the data by systematically investigating a set of transformations which is simple, but nevertheless very flexible, and represents a wide range of functional forms.

The results of the FP approach and of the traditional approach based on categorized covariates are compared in investigating the functional influence of seven standard prognostic factors in a prospective study on node-positive breast cancer patients of the German Breast Cancer Study Group (Schumacher et al, 1994). It will be shown that the FP approach can provide clearer insight into the nature of the relationship between the values of the factors and the risk of a new event than usual approaches. This is particularly so in our study for the effect of the patients' age.

### MATERIALS AND METHODS

The principal eligibility criterion was a histologically verified primary breast cancer of stage T1a-3aN+M0. Primary local treatment was by a modified radical mastectomy (Patey) with en bloc axillary dissection with at least six identifiable lymph nodes. Patients should not be older than 65 years of age and should present with a Karnofsky index of at least 60. The study was designed as a Comprehensive Cohort Study (CCS), i.e. patients who satisfy the entry criteria are informed about the study and are asked whether they agree to be randomized (Schmoor et al, 1996). If so, they are randomized to a treatment, otherwise, they or their physicians choose their preferred treatment. Randomized and non-randomized patients were included in the present analysis of prognostic factors.

The study has a 2 × 2 factorial design with the four adjuvant treatment arms: (A) 3 × CMF; (B) 3 × CMF + TAM; (C) 6 × CMF; and (D) 6 × CMF + TAM. Although we could randomize shortly after the operation, we aimed to randomize as late as possible, presumably after basic chemotherapy of three cycles, to increase compliance. Following the statements of the National Cancer Institute consensus conference, the protocol was modified in December 1986 with premenopausal patients only randomized between treatment arms A and C (Consensus Conference, 1985). Chemotherapy was administered using the modified Bonnadonna CMF scheme consisting of 500 mg $m^{-2}$ cyclophosphamide, 40 mg $m^{-2}$ methotrexate and 600 mg $m^{-2}$ fluorouracil i.v. on day 1 and 8 of a 4-week treatment period. Hormonal treatment consisted of a daily dose of 3 × 10 mg tamoxifen p.o. over 2 years starting after the third cycle of CMF. Major prognostic factors evaluated in the trial were patient's age, menopausal status, tumour size, oestrogen and progesterone receptor status, tumour grading according to Bloom and Richardson, histological tumour type and number of involved lymph nodes. Histopathologic classification was re-examined, and grading was performed centrally by one pathologist for all cases. Hormone receptor content, both ER and PR, was measured biochemically by a dextran-coated charcoal method and classified as positive if the respective value was equal or greater than 20 fmol $mg^{-1}$. Quality control for the hormone receptor analysis was performed centrally. Patients were followed up at regular intervals to ensure detection of any kind of recurrence at the earliest time possible. For more details of the study see Schumacher et al (1994).

The primary end point was tumour recurrence or death of a patient. RFS was defined as time from mastectomy to the first occurrence of either locoregional or distant recurrence, contralateral tumour, secondary tumour or death; overall survival (OS) as time from operation to death. Recurrence-free and overall survival rates were estimated by the Kaplan–Meier product limit method. The Cox proportional hazards model was used to investigate simultaneously the influence of several factors on the survival times (Byar, 1984). The risk function for a patient with values ($Z_1$, $Z_2$, …, $Z_k$) of the prognostic factors can be written as

$$\lambda_0(t)\exp(\beta_1 Z_1 + \beta_2 Z_2 + \ldots + \beta_k Z_k)$$

**Table 1** Patient characteristics with respect to prognostic factors. Values in parentheses are the 25th, 50th (median) and 75th centiles of the data for the variable in question

| Variable | Category | No. | % |
|---|---|---|---|
| Age, years | ≤ 45 | 153 | 22.3 |
| (46, 53, 61) | 46–60 | 345 | 50.3 |
| | > 60 | 188 | 27.4 |
| Menopausal state | pre | 290 | 42.3 |
| | post | 396 | 57.7 |
| Tumour size, mm | ≤ 20 | 180 | 26.2 |
| (20,25,35) | 21–30 | 287 | 41.8 |
| | > 30 | 219 | 31.9 |
| Tumour grading | I | 81 | 11.8 |
| | II | 444 | 64.7 |
| | III | 161 | 23.5 |
| No. of involved nodes | 1–3 | 376 | 54.8 |
| (1, 3, 7) | 4–9 | 207 | 30.2 |
| | ≥ 10 | 103 | 15.0 |
| Progesterone receptor, | < 20 | 269 | 39.2 |
| fmol (7,33,132) | ≥ 20 | 417 | 60.8 |
| Oestrogen receptor, fmol | < 20 | 262 | 38.2 |
| (8,36,115) | ≥ 20 | 424 | 61.8 |

© Cancer Research Campaign 1999

where $\lambda_0(t)$ is an unspecified baseline hazard function. The coefficients $\beta_1, \beta_2, \ldots, \beta_k$, which are estimated from the data, represent the influence of the different factors. To investigate the effect of different prognostic subgroups the variables are defined as indicators for the respective subgroup. The relative risk of the subgroup defined by $Z_i$ in relation to the reference group is then given by $\exp(\beta_i)$. All analyses investigating the influence of prognostic factors are adjusted for hormonal treatment, whereas the duration of CMF was not included in the model. All *P*-values are based on the likelihood ratio test. To develop a more parsimonious model, including only variables with an influence on the outcome, we used backward elimination (selection level 0.05). To investigate the functional relationship of a continuous factor on RFS and OS, we used two usual approaches (a) and (b), and a new approach (c):

a. The factor was included in the model as originally measured, i.e. a log linear relationship between the factor and the risk of death is assumed. This will be called the 'linear approach'.
b. The factor was categorized into two or three groups according to the predefined cutpoints used in the primary analysis of the randomized part of the trial (Schumacher et al, 1994), i.e. a step functional relationship between the factor and the risk of death is assumed. This will be called the 'step approach'.
c. The recent fractional polynomials approach was used, where among a defined class of transformations and under some constraints concerning model complexity the best fitting functional form is selected (Royston and Altman, 1994). For details see the Appendix. This will be called the 'FP approach'.

Sauerbrei and Royston (1999) proposed modifications of the multivariable FP version to incorporate basic medical knowledge of the types of relationship to be expected between certain predictors and risk. For one variable only, the number of positive lymph nodes, they decided that the relationship should be modelled as a function which always increased but which levelled off at a high number of positive nodes (technically known as a monotonic function with an asymptote). We adopted the same approach and used the same simple primary transformation as Sauerbrei and Royston (1999), namely $\exp(-0.12 \times \text{nodes})$. The factor –0.12 was estimated from the data.

We start with a univariate analysis of the prognostic factors. We then investigate their influence simultaneously in multivariable models.

For checking the FP functional form we use generalized additive models (GAMs; Hastie and Tibshirani, 1990). For a brief description of this flexible approach see the Appendix. For the three approaches (a), (b) and (c) the estimated relative risks are standardized in the sense that the baseline category from the step approach is taken as a reference with a relative risk of 1.

### RESULTS

From July 1984 to December 1989, 41 centres recruited 720 patients, of whom about two-thirds were randomized. Complete data of the seven standard factors as given in Table 1 were available for 686 (95.3%) patients, who are taken as the basic patient population in this paper. After a median follow-up time of nearly 5 years, 299 events for RFS and 171 deaths were observed. Because of the patients' preference in the non-randomized part, and because of the change in protocol concerning premenopausal patients, only about a third of the patients received hormonal treatment. Age and menopausal status had a strong influence on whether this therapy was administered. As stated earlier we adjust all analyses for hormonal treatment.

### Univariate models for RFS

In Table 2 we give the results of investigating the functional influence of the five continuous prognostic factors in univariate models, adjusted only for hormonal treatment. We list the *P*-values for two model comparisons:

a. a linear effect versus no effect
b. the effect of a second degree fractional polynomial versus a linear effect.

Comparison (a) represents the simplest approach for modelling a continuous predictor without grouping the values. The first column of Table 2 shows the strong influence of tumour size, number of involved lymph nodes and PR, but age and ER seem not to be prognostic. Comparison (b) is part of our strategy to detect and model non-linear relationships. By contrast with (a), for age and ER there are highly significant ($P$ < 0.001) differences between the second degree FP and the linear model, showing that these factors have a strong non-linear effect on RES. The influence of PR is also seen to be non-linear. The effect of exp(–0.12*nodes) is linear. Since the improvement in fit due to the exponential transformation of nodes compared with a linear model is highly significant (Sauerbrei and Royston, 1999), the effect of nodes is also found to be strongly non-linear. With the step approach, only age is not significant; the four other continuous variables are significant at the 1% level. Among the categorical variables, tumour grade shows a strong effect whereas the effect of menopausal status is non-significant.

**Table 2** *P*-values for univariate FP analyses of continuous covariates, all adjusted for hormonal treatment

| | Recurrence-free survival (RFS) | | Overall survival (OS) | |
|---|---|---|---|---|
| | Linear vs none | FP (2) vs linear | Linear vs none | FP (2) vs linear |
| Age | 0.93 | < 0.001 | 0.56 | 0.75 |
| Tumour size | < 0.001 | 0.23 | <0.001 | 0.62 |
| Transformed nodes[a] | <0.001 | 0.23 | <0.001 | 0.77 |
| Progesterone receptor | <0.001 | <0.001 | <0.001 | <0.001 |
| Oestrogen receptor | 0.063 | <0.001 | 0.023 | <0.001 |

[a]Primary transformation exp(–0.12 × nodes), see end of Section 2 and Appendix.

 *© Cancer Research Campaign 1999*

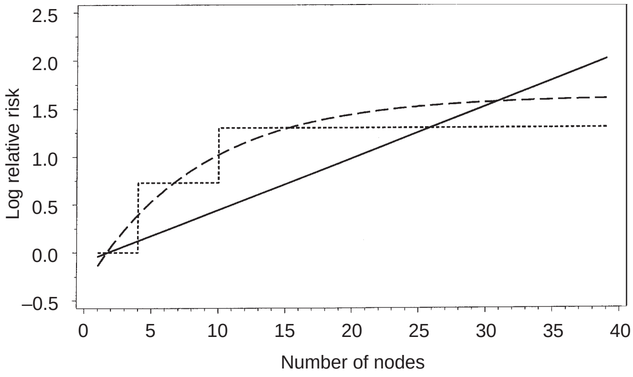

**Figure 1** Postulated functional influence of the number of positive lymph nodes (PN) on the log relative risk of recurrence-free survival in multivariable models according to three modelling approaches. To enable comparison between models, each of the fitted curves has been standardized by subtracting an appropriate constant. The functions plotted are (**A**) 0.054 × PN – 0.0925; (**B**) step function with log relative risks 0, 0.731, 1.301; (**C**) –1.9812 × exp(–0.12 × PN) + 1.6203. (—) Linear function; (- - -) step function, (– – –) fractional polynomials

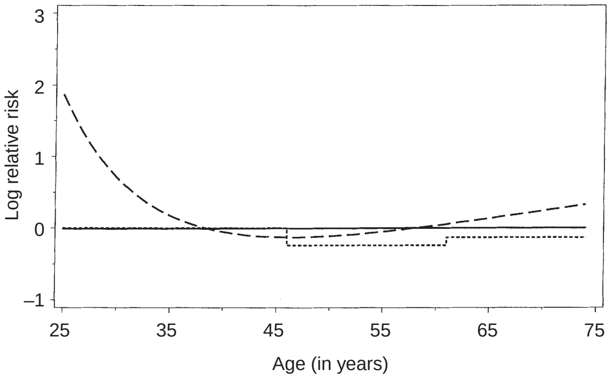

**Figure 2** Postulated functional influence of age on the log relative risk of recurrence-free survival (details as for Figure 1). The functions plotted are (**A**) 0.000383×age –0.0151; (**B**) step function with log relative risks 0, –0.24, –0.13; (**C**) 1.7422 × $(age/50)^{-2}$ – 7.8179 × $(age/50)^{-0.5}$ + 5.884. (——) Linear function, (- - -) step function, (– – –) fractional polynomials

### Multivariable models for RFS

In Table 3 we give the final multivariable models for recurrence-free survival. With the usual approaches based on a linear function or on categorized variables and with backward elimination as the selection procedure, grade, PR and number of positive lymph nodes show a prognostic effect. In Figure 1 we present the estimated relative risk of the lymph nodes component from the final models. The increase in relative risk can be seen. Patients with at least ten nodes have a significantly worse prognosis than those with 4–9 nodes. The linear model may underestimate the effect for a small number of nodes, whereas it may substantially overestimate it for a very large number.

As well as the three factors selected with the linear and step approaches, the final model based on fractional polynomials indicates a strong effect of age on RFS. Interpretation of the parameter estimates can best be demonstrated graphically. In Figure 1 it is shown that the FP approach for nodes implies an increased relative risk with an asymptote of about a fivefold risk for a large number of positive nodes. The function fitted by the step approach can be seen as a reasonable approximation.

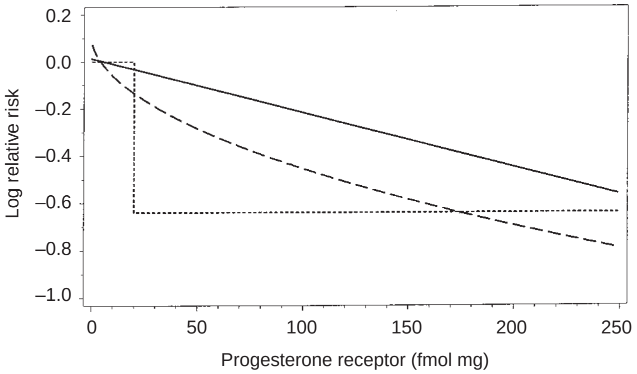

**Figure 3** Postulated functional influence of progesterone receptor (PR) on the log relative risk of recurrence-free survival (details as for Figure 1). The functions plotted are (**A**) –0.0023 × PR+0.0125; (**B**) step function with log relative risks 0, –0.639; (**C**) –0.0582 × $(PR+1)^{0.5}$ + 0.131. (——) Linear function, (---) step function, (– – –) fractional polynomials

**Table 3** Final multivariable models for recurrence-free survival, adjusted for hormonal treatment (*P*-values from likelihood ratio tests)

**a) Linear approach**

| Variables | β | SE | *P*-value |
|---|---|---|---|
| Grade | | | |
| 1 | 0 | – | – |
| 2 or 3 | 0.687 | 0.247 | 0.002 |
| No. of nodes | 0.054 | 0.007 | <0.001 |
| Progesterone receptor (PR) | –0.0023 | 0.0006 | <0.001 |

**b) Step approach**

| Variable with categories | β | SE | *P*-value |
|---|---|---|---|
| Grade | | | |
| 1 | 0 | – | – |
| 2 or 3 | 0.547 | 0.250 | 0.019 |
| No. of nodes | | | |
| 1–3 | 0 | – | – |
| 4–9 | 0.731 | 0.134 | <0.001 |
| ≥ 10 | 1.301 | 0.153 | <0.001 |
| Progesterone receptor (PR) | | | |
| < 20 | 0 | – | – |
| ≥ 20 | –0.639 | 0.120 | <0.001 |

**c) FP approach**

| Variable with transformation | β | SE | *P*-value |
|---|---|---|---|
| $(age/50)^{-2}$ | 1.742 | 0.330 | <0.001 |
| $(age/50)^{-0.5}$ | –7.818 | 1.749 | <0.001 |
| Grade | | | |
| 1 | 0 | – | – |
| 2 or 3 | 0.517 | 0.249 | 0.026 |
| Transformed nodes[a] | –1.981 | 0.227 | <0.001 |
| Transformed progesterone receptor[b] | –0.0582 | 0.0111 | <0.001 |

[a]Primary transformation exp(–0.12 * nodes), see end of section 2 and Appendix. [b]$(PR + 1)^{0.5}$.

In Figure 2 we give the estimated functional relationships for the age effect which demonstrates that the linear approach does not show any effect. This could be expected, as age was not significant in the multivariable model and was only added to the final model to estimate its effect. The FP approach indicates that younger patients, up to an age of about 40, have a highly increased risk, and

*© Cancer Research Campaign 1999*

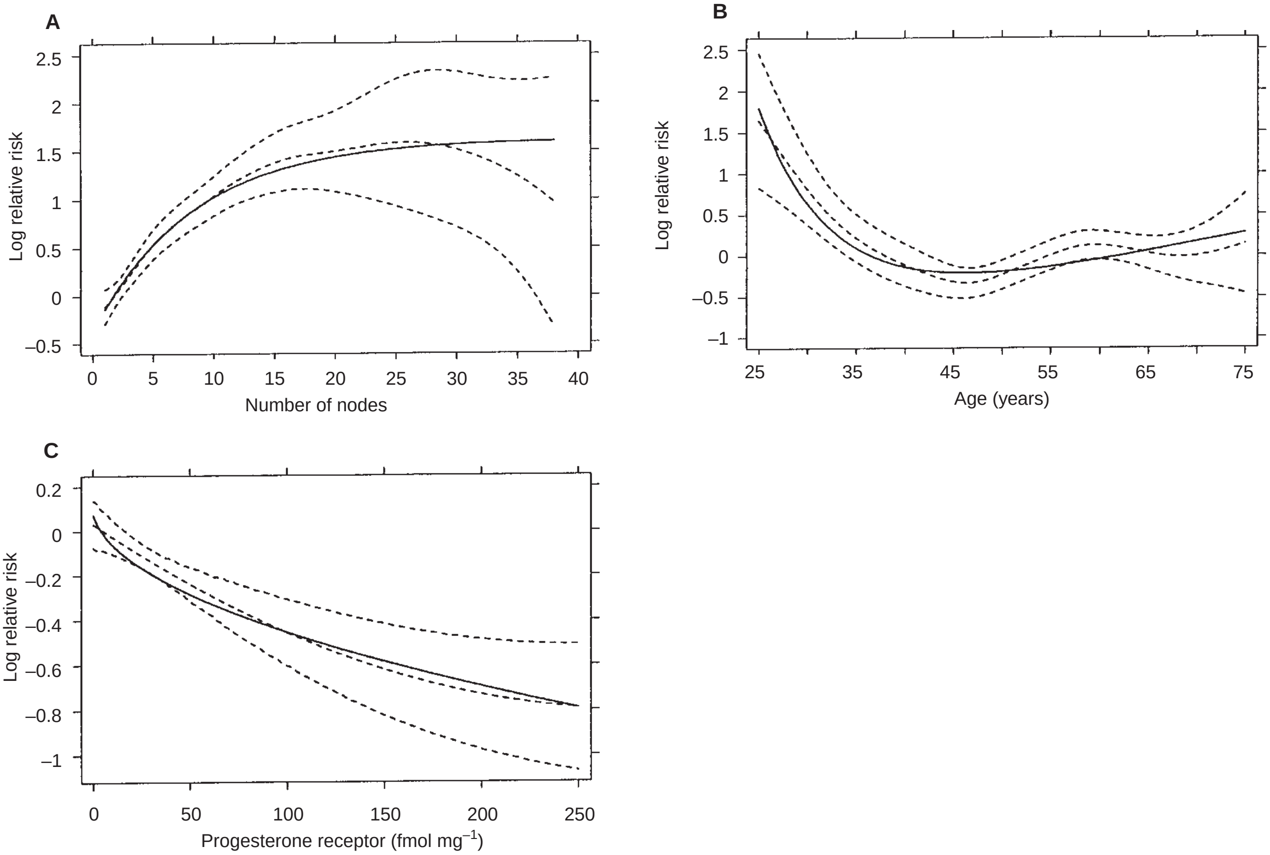

**Figure 4** Check of the postulated FP functions for recurrence-free survival from the final model using generalized additive models (GAM). —–, FP; - - -, GAM with 95% pointwise confidence interval. (**A**) number of positive lymph nodes; (**B**) age; (**C**) progesterone receptor that after a fairly constant period between 40 and 55 years the risk increases again. In the step approach we added the non-significant age effect to the corresponding final model in order to demonstrate the behaviour of this procedure. The effect is very small, perhaps as a result of choosing the predefined cutpoint of 45 years for the young age group.

In Figure 3 we show the estimated functional relationships for PR from the three modelling approaches. In contrast to the linear approach, the FP approach shows a steep decrease for very small values.

The effect of grade is similar in the different models with a better prognosis for grade 1 patients and no differentiation between grade 2 and grade 3. To check the suggested functional form of the final model, in Figure 4 we plot the FP function and the fitted GAM curve with pointwise 95% confidence limits for all three continuous factors. The GAM curve was fitted for one variable, with the other variables and corresponding FP functions fixed as given in the final model for RFS.

In each case the confidence limits for the GAM curve include the fitted FP curve, which suggests that the two models are statistically compatible. This provides reassurance that the FP approach has not missed some important feature of the relationship.

### Models for OS

In univariate analyses of OS, age has no apparent effect but the four other continuous variables are all significant (Table 2). The effect seems to be linear for tumour size only. In multivariable analysis with the FP approach, only the number of nodes and PR entered the final model, whose prognostic index is given by

$$-2.176 \times \exp(-0.12 \times \text{nodes}) - 0.127 \times (\text{progesterone} + 1)^{0.5}.$$

The graphs for these two factors show that the functions resemble those for RFS and are supported by the model-checking method using GAMs (data not shown). With the step approach, tumour size was also included in the model and had a weak effect. With the linear approach, tumour size and grade were included in the model in addition to the two dominating factors (number of nodes, progesterone receptor).

### DISCUSSION

Prognostic factors play an important role in the management of breast cancer and in clinical research. The role of several standard factors has been investigated in hundreds of papers and is still the subject of controversy. Some of the important reasons for the situation concern statistical aspects such as small sample size or inadequate ways of modelling complex multivariable relationship (Simon and Altman, 1994).

Categorization of continuous variables is often used and certainly has several advantages, including simplicity of the final model and robustness to outliers or to problems of data quality. Not least, the analysis is cost-effective in terms of the time, effort and skill required to undertake it. If several categories are considered in

*© Cancer Research Campaign 1999*

**Table 4** Fractional polynomial models for the effect of age on recurrence-free survival. The best-fitting first and second degree models are indicated by underlining

| Fractional polynomials | | | | | | | | | | |
|---|---|---|---|---|---|---|---|---|---|---|
| First-degree | | Second-degree | | | | | | | | |
| Power | Model | Powers | | Model | Powers | | Model | Powers | | Model |
| *p* | $\chi^2$ | *p* | *q* | $\chi^2$ | *p* | *q* | $\chi^2$ | *p* | *q* | $\chi^2$ |
| –2 | 6.41 | –2 | –2 | 17.09 | –1 | 1 | 15.56 | 0 | 2 | 11.45 |
| –1 | 3.39 | –2 | –1 | 17.57 | –1 | 2 | 13.99 | 0 | 3 | 9.61 |
| –0.5 | 2.32 | –2 | –0.5 | 17.61 | –1 | 3 | 12.37 | 0.5 | 0.5 | 13.37 |
| 0 | 1.53 | –2 | 0 | 17.52 | –0.5 | –0.5 | 16.82 | 0.5 | 1 | 12.29 |
| 0.5 | 0.97 | –2 | 0.5 | 17.30 | –0.5 | 0 | 16.18 | 0.5 | 2 | 10.19 |
| 1 | 0.58 | –2 | 1 | 16.97 | –0.5 | 0.5 | 15.41 | 0.5 | 3 | 8.32 |
| 2 | 0.17 | –2 | 2 | 16.04 | –0.5 | 1 | 14.55 | 1 | 1 | 11.14 |
| 3 | 0.03 | –2 | 3 | 14.91 | –0.5 | 2 | 12.74 | 1 | 2 | 8.99 |
| | | –1 | –1 | 17.58 | –0.5 | 3 | 10.98 | 1 | 3 | 7.15 |
| | | –1 | –0.5 | 17.30 | 0 | 0 | 15.36 | 2 | 2 | 6.87 |
| | | –1 | 0 | 16.85 | 0 | 0.5 | 14.43 | 2 | 3 | 5.17 |
| | | –1 | 0.5 | 16.25 | 0 | 1 | 13.44 | 3 | 3 | 3.67 |

See Appendix for further details.

the analysis, a test for trend may be used to assess a monotonic effect of a factor. However, there are several well-known drawbacks. The final model is a risk step-function which changes suddenly as one crosses a cutpoint. Because cutpoints are usually not 'naturally' given, and because there is often no consensus as to a sensible cutpoint, investigators may be tempted to search for the 'optimal' cutpoint in their study, 'optimal' meaning 'most significant'. The severe problems of this approach are a substantial overestimation of the effect size, too small *P*-values, and loss of information because of categorization as demonstrated by Altman et al (1994). Such an approach will lead to different cutpoints in the single studies, which makes comparing the results of several studies nearly impossible. Even if the cutpoints are given a priori, considerable information may be lost. A further problem is terminal digit preference, which is common with certain measurements (e.g. tumour size 10, 20 mm). Defining a group as 'smaller than *x*' rather than 'smaller than or equal to *x*' may give substantially different results. The effect of terminal digits is much less pronounced if the data are not categorized. Using the data in continuous form as originally measured, a straight line is the most common assumption used to describe the functional influence of a prognostic factor on the outcome. If the assumption is wrong, there are serious consequences for the inclusion of a prognostic factor in the final model.

In accordance with the literature, the number of positive lymph nodes is the factor with the strongest influence on the two survival criteria. With all types of analyses the significant effect can be demonstrated, but Figure 1 shows the differences. The step-function model jumps at the predefined cutpoints 3 and 10, but can be seen as a rough approximation to the form from the final FP model. The linear model may underestimate the effect for a small number of nodes, whereas it overestimates this effect for a very large number, e.g. 40 or more. Based on current medical knowledge we forced the functional influence of the number of nodes to be monotonic with an asymptote, which we achieved using an initial exponential transformation (for details see Sauerbrei and Royston, 1998).

Furthermore, PR is included in all final models for RFS and OS with a highly significant reduced risk for increasing concentration whereas ER never showed an effect in the multivariable context. This result confirms the earlier result in a subpopulation of the patients analysed here (Schumacher et al, 1994). The FP function shows a steep decrease for very small values; such a function may explain the chosen cutpoints (usually between 5 fmol mg$^{-1}$ and 20 fmol mg$^{-1}$) to define receptor negativity for the investigation of the prognostic value of receptor measurements as a binary variable.

For recurrence-free survival tumour grade is also included in the final models of all three types of analysis. Grades 2 and 3 are always indicating little or no difference between them, but patients with that grade 1 tumour have a substantially reduced risk. Combining grade 2 and 3 is in agreement with results from Cummings et al (1995) and Pichon et al (1996), who found large differences between grade 1 and 2 in a model adjusting for axillary node status, tumour size, age and ER, whereas the risk increased only slightly from grade 2 to grade 3. Grade was also established as one of the three factors in the Nottingham Prognostic Index (Galea, 1992), which seems to be the only prognostic classification scheme capable of being validated more than once (Brown et al, 1993, Balslev et al, 1994, Sauerbrei et al, 1997). Generally, the issues of which grading schemes to use and the importance or otherwise of grade as a prognostic factor in a multivariable context are controversial (Elston and Ellis, 1991; Schumacher et al, 1993). For overall survival the importance of grade could not be demonstrated. Grade only entered the final model with the linear approach, but with categories 1 and 2 combined. This model was the only one which indicated a strong effect of tumour size ($P < 0.01$). Tumour size also entered ($P = 0.04$) the final model for OS in the analysis based on categorized variables.

The FP approach clearly demonstrates a strong non-linear effect of age on RFS. As Figure 2 shows there seems to be a steep increase in the hazard for patients younger than 40 years. The model implies that the risk is similar for patients between 40 and 60 years. Age is an independent prognostic factor in addition to the number of positive lymph nodes, tumour grade and PR, which contrasts with the conclusions of Kollias et al (1997) who investigated the effect of age in different subgroups defined by the Nottingham Prognostic Index. Besides the standard factors investigated here, there seem to be other factors that make breast carcinomas in young women different and that may be responsible for

© Cancer Research Campaign 1999

the bad prognosis (Walker et al, 1996). With the two usual analysis approaches we could not demonstrate an effect of age on RFS. This is not surprising for the linear approach because the effect seems to be strongly non-linear. With the step approach we could not establish the age effect because we used the categories based on predefined cutpoints of 45 and 60 years (Schumacher et al, 1994). This decision was intended to avoid well-known problems with the optimal cutpoints approach (Altman et al, 1994). Our estimated FP function seems to explain the current discussion about several cutpoints for young age which range at least from 33 (Rochefordiere et al, 1993) to 45 (Crowe et al, 1994; Collett et al, 1996). Moreover, our results give some indication that the effect of age is sometimes not established because of differences in statistical approaches. Besides the three different types of postulated functions, the other factors studied may have a strong influence on the final conclusions on the age effect as an independent prognostic factor in a multivariable context. Because of the large variation in competing factors between different studies a final conclusion cannot be reached at present. As an example, Chung et al (1996) concluded that ‘women 40 years of age and younger have a worse 5 year cancer-specific survival than their older counterparts’. They included 3722 women in their study, used age in 10-year intervals and analysed the data in three groups by stage. In a study on 2879 women, Kollias et al (1997) compared patients aged < 35 years with older age groups, stratified by three groups defined by the Nottingham Prognostic Index, and concluded that ‘age itself had no influence on the prognosis of the individual’. Pichon et al (1996) had complete data for 1665 patients on the covariates grade, number of axillary nodes, tumour size, ER status, TNM stage, menopausal status and age. For disease-free and metastasis-free survival they found a strong age effect (relative risk 2.8) in a multivariable model by comparing patients ≤ 35 years with those older than 35. However, as with our analysis, age did not enter the final model for overall survival.

An important step for an improvement in summarizing information from prognostic factors would be a widely accepted standard prognostic model. We see the Nottingham Prognostic Index as the most often validated and accepted classification scheme. As recently published for node-negative patients where, in addition to changes to the weights of the component of the NPI, in one of our proposals only age was added to the NPI (Sauerbrei et al, 1997), we see the results from the NPI and our proposals as a starting point for an urgently needed accepted and sensible description of the influence of standard factors.

FPs may also be used to investigate a possible treatment/covariate interaction. After fitting a single model with a separate FP curve for each treatment group, and with the rest of the multivariable model unchanged, a substantial difference in the type of FP curve for each group may indicate of a treatment/covariate interaction. Alternatively, the original FP transformation, such as square root, may be retained and a separate slope estimated for the transformed variable in each group. A significant difference in the slopes may be evidence of an interaction. However, some methodological issues connected with this way of analysing treatment/covariate interactions are unresolved.

We have proposed a multivariable approach to analysis which avoids categorization and retains continuous variables as continuous. This avoids loss of information and postulates functional forms that are more realistic than step functions. If grouping is required for clinical reasons, e.g. to define criteria for risk adapted trials, it can be done in the usual way, but based on a more sensible prognostic index suggested by the shape of the FP curve. Given the FP model, we checked the proposed form for each continuous variable against that from generalized additive models. Because the final model of a GAM approach cannot be given as a concise mathematical expression, this alternative makes clinical interpretation and application very difficult. However, it can be seen as an advantageous approach to demonstrate that the proposed FP function does exhibit essentially all the important information present in the data. Of course, any postulated models have to be validated in new studies.

### REFERENCES

Altman DG, Lausen B, Sauerbrei W and Schumacher M (1994) Dangers of using ‘optimal’ cutpoints in the evaluation of prognostic factors. *J Natl Cancer Inst* **86**: 829–835

Balslev I, Axelsson CK, Zedeler K, Rasmussen BB, Carstensen B and Mouridsen HT (1994) The Nottingham Prognostic Index applied to 9149 patients from the studies of the Danish Breast Cancer Cooperative Group (DBCG). *Breast Cancer Res Treat* **32**: 281–290

Brown JM, Benson EA and Jones M (1993) Confirmation of a long-term prognostic index in breast cancer. *Breast* **2**: 144–147

Byar DP (1984) Identification of prognostic factors. In *Cancer Clinical Trials – Methods and Practice*, Buyse ME, Staquet MJ, Sylvester RJ (eds), pp. 423–443. Oxford University Press, Oxford

Chung M, Chang HR, Bland KI and Wanebo HJ (1996) Younger women with breast carcinoma have a poorer prognosis than older women. *Cancer* **77**: 97–103

Collett K, Hartveit F, Skjaerven R and Maehle BO (1996) Prognostic role of oestrogen and progesterone receptors in patients with breast cancer: relation to age and lymph node status. *J Clin Pathol* **49**: 920–925

Crowe JP, Gordon NH, Shenk RR, Zollinger RM Jr, Brumberg DJ and Shuck JM (1994) Age does not predict breast cancer outcome (with discussion). *Arch Surg* **129**: 483–488

Cox DR (1972) Regression models and life tables (with discussion). *J R Stat Soc B* **34**: 187–220

Cummings MC, Wright RG, Furnival CM, Bain CJ and Siskind V (1995) The feasibility of retrospective grading of breast cancer histology slides derived from multiple pathology services. *Breast* **4**: 179–182

Elston CW and Ellis IO (1991) Pathological prognostic factors in breast cancer: the value of histologic grade in breast cancer: experience from a large study with long-term follow-up. *Histopathology* **19**: 403–410

Galea MH, Blamey RW, Elston CE and Ellis IO (1992) The Nottingham Prognostic Index in primary breast cancer. *Breast Cancer Res Treat* **22**: 207–219

Hastie TJ and Tibshirani RJ (1990) *Generalized Additive Models*. Chapman and Hall: New York

Kollias J, Elston CW, Ellis IO, Robertson JFR and Blamey RW (1997) Early-onset breast cancer: histopathological and prognostic considerations. *Br J Cancer* **75**: 1318–1323

Lagakos SW (1988) The loss in efficiency from misspecifying covariates in proportional hazards regression models. *Biometrika* **75**: 156–160

Palta M and Amini SB (1985) Consideration of covariates and stratification in sample size determination for survival time studies. *J Chronic Dis* **38**: 801–809

Pichon MF, Broet P, Magdelenat H, Delarue JC, Spyratos F, Basuyau JP, Saez S, Rallet A, Courriere P, Millon R and Asselain B (1996) Prognostic value of steroid receptors after long-term follow-up of 2257 operable breast cancers. *Br J Cancer* **73**: 1545–1551

de la Rochefordiere A, Asselain B, Campana F, Scholl SM, Fenton J, Vilcoq JR, Durand JC, Pouillart P, Magdelenat H and Fourquet A (1993) Age as prognostic factor in premenopausal breast carcinoma. *Lancet* **341**: 1039–1043

Royston P and Altman DG (1994) Regression using fractional polynomials of continuous covariates: parsimonious parametric modelling (with disc). *Applied Statistics* **43**: 429–467

Sauerbrei W and Royston P (1999) Building multivariable prognostic and diagnostic models: transformation of the predictors using fractional polynomials. *J R Stat Soc A* **162**: 71–94

Sauerbrei W, Hübner K, Schmoor C, Schumacher M for the German Breast Cancer Study Group (1997) Validation of existing and development of new prognostic classification schemes in node negative breast cancer. *Breast Cancer Res Treat* **42**: 149–163 (Erratum in *Breast Cancer Res Treat 1998*; **43**: 191–192)

Schmoor C and Schumacher M (1997) Effects of covariate omission and categorization when analysing randomized trials with the Cox model. *Stat Med* **16**: 225–237

 © Cancer Research Campaign 1999

Schmoor C, Olschewski M and Schumacher M (1996) Randomized and non-randomized patients in clinical trials: experiences with comprehensive cohort studies. *Stat Med* **15**: 263–271

Schumacher M, Schmoor C, Sauerbrei W, Schauer A, Ummenhofer L, Gatzemeier W, Rauschecker H for the German Breast Cancer Study Group (1993) The prognostic effect of histological tumour grade in node-negative breast cancer patients. *Breast Cancer Res Treat* **25**: 235–245

Schumacher M, Bastert G, Bojar H, Hübner K, Olschewski M, Sauerbrei W, Schmoor C, Beyerle C, Neumann RLA, Rauschecker HF for the Breast Cancer Study Group (1994) Randomized 2×2 trial evaluating hormonal treatment and the duration of chemotherapy in node-positive breast cancer patients. *J Clin Oncol* **12**: 2086–2093

Simon R and Altman DG (1994) Statistical aspects of prognostic factor studies in oncology. *Br J Cancer* **69**: 979–985

Walker RA, Lees E, Webb MB and Dearing SJ (1996) Breast carcinomas occurring in young women (<35 years) are different. *Br J Cancer* **74**: 1796–1800

### APPENDIX

### Fractional polynomials

Suppose we wish to construct a Cox proportional-hazards regression model with which to predict the relative risk of disease recurrence in terms of *age* (the patient's age, regarded as a continuous variable). A possible non-linear function with which to represent the relationship is a quadratic polynomial model in *age*, as follows:

$$\log \text{RR} = a + b \times age^{1} + c \times age^{2}$$

Quadratic polynomials, while undoubtedly useful, suffer various disadvantages as regression models, including a limited range of possible curve shapes. Generalizations of polynomials known as fractional polynomials (Royston and Altman, 1994) are obtained by replacing the whole-number powers 1 and 2 by less restrictive numbers, $p$ and $q$:

$$\log \text{RR} = a + b \times age^{p} + c \times age^{q}$$

The powers $p$ and $q$ are not allowed to be completely free, but are chosen from the small set –2, –1, –0.5, 0, 0.5, 1, 2, 3. The 'power' 0 represents a natural logarithmic transformation, so that $age^0$ is defined as $\log_e(age)$. This simple extension generates a considerable range of new curve shapes which are useful in data analysis (Royston and Altman, 1994). For example, a fractional polynomial with powers (0, 2), that is with $p = 0$ and $q = 2$, is

$$a + b \times \log(age) + c \times age^{2}$$

When $p = q$, the complete family of curves also includes functions of the form

$$\log \text{RR} = a + b \times age^{p} + c \times age^{p} \times \log(age).$$

These are known as 'repeated powers models' because they represent the mathematical limit attained when $p$ and $q$ approach each other arbitrarily closely.

First-degree fractional polynomials are simple, familiar power transformations of the predictor. They include reciprocal, logarithmic, square root and square transformations. They are guaranteed to produce fitted curves that are 'monotonic', that is, that always increase or always decrease as the predictor increases. Second-degree fractional polynomial curves have at most one turning point (minimum or maximum) which may or may not lie within the observed range of predictor values. However, some second-degree curves are monotonic, and it is straightforward to determine this by inspecting the signs of the powers $p$, $q$ and the coefficients b, c.

Higher degree fractional polynomials have greater flexibility still, but we do not generally recommend their use in data analysis and did not use them in the analyses in the present paper.

We now consider fractional polynomial model selection. A fractional polynomial of first-degree is of the form $a+b\ age^{p}$. The special case $p = 1$ corresponds to a straight line is our preferred choice unless there is convincing evidence that it does not fit the data adequately. We adopt a forward selection approach to look for such evidence. For a given data-set, we fit the eight regression models $a + b\ x$ with $x = age^{-2}$, $x = age^{-1}$, …, $x = age^{3}$ in turn and find the value of $p$ which maximizes the likelihood. This is equivalent to maximizing the model $\chi^2$ statistic or to minimizing the deviance, which is defined as minus twice the log likelihood. The hypothesis that $p = 1$ is tested against $p \neq 1$ using a $\chi^2$ test with one degree of freedom (df). If the test is significant at the 5% level, the linear model is rejected. Next we look for evidence of a more complex curve shape by finding the best-fitting second-degree fractional polynomial. This involves searching for the powers $p$ and $q$ which maximize the model $\chi^2$ among the 36 two-term models of the form $a + b\ age^{p} + c\ age^{q}$ and $a + b\ age^{p} + c\ age^{p} \log(age)$. The required hypothesis test of second-degree versus first-degree fractional polynomial now has 2 df, one for estimating the regression coefficient c and one for estimating the power $q$. With no prior knowledge about the shape of the relationship we again perform the test at the 5% significance level. If we want to force the relation to be monotonic, for example because of prior medical knowledge, we prefer a first-degree fractional polynomial unless there is strong contrary evidence. We may therefore test at a more rigorous significance level such as 1% to reduce the risk of choosing an inappropriate model.

To illustrate the process, Table 4 gives the results of fitting fractional polynomials with *age* as the only predictor of recurrence-free survival. The $\chi^2$ value for a straight line model is 0.58 (see $p = 1$ in Table 4), which gives no indication of a linear prognostic effect of *age* ($P = 0.4$). However, the best-fitting first degree fractional polynomial has power $p = -2$ and a model $\chi^2$ of 6.41, indicating a significant non-linear effect (deviance difference = 5.83 on 1 df, $P = 0.02$). The best second-degree fractional polynomial has powers $p = -2$, $q = -0.5$ and a model $\chi^2$ of 17.61. The fit is convincingly better than that of the first-degree model (deviance difference = 11.20 on 2 df, $P = 0.004$). Note that several second-degree models have $\chi^2$ values not much smaller than that of the best model. This is a typical feature of such an analysis and is similar to deciding in favour of the best model in all-subsets variable selection. It implies some arbitrariness in the final model. For example, there are seven models with $\chi^2 > 17$, but their fitted curves turn out to be much alike.

When the model may contain several continuous predictors (the usual situation with prognostic factor studies), it is impracticable to search for the best model among all possible combinations of powers for each of the predictors. Instead we use a procedure based on backward elimination of uninfluential variables and selection of the best fractional polynomial for each predictor in turn, adjusting for the other predictors. Details are given by Sauerbrei and Royston (1999).

### Generalized additive models

Generalized additive models or GAMs (Hastie and Tibshirani, 1990) provide a flexible approach to modelling curved regression relationships. The conventional multiple linear regression function

© Cancer Research Campaign 1999

$\beta_1 Z_1 + \beta_2 Z_2 + \ldots + \beta_k Z_k$ is replaced with a sum of functions, $f(Z_1) + f(Z_2) + \ldots + f(Z_k)$. Each 'f' is a cubic smoothing spline (a special type of cubic polynomial) whose complexity is determined by its 'equivalent degrees of freedom' or edf. A higher edf gives a more flexible family of curve shapes. Rather than fit a GAM for all the prognostic factors simultaneously we compared a GAM (5 edf) with the FP for each predictor individually, keeping the rest of the original FP model. The fitted GAM curves and pointwise 95% confidence limits (as a guide to the precision of the curve) were plotted for each predictor.

 *© Cancer Research Campaign 1999*
